# Supplementary material for: Social Return on Investment (SROI) Evaluation of Citizens Advice on Prescription: A Whole-Systems Approach to Mitigating Poverty and Improving Wellbeing
Source: Int J Environ Res Public Health. 2025 Feb 17;22(2):301. doi: 10.3390/ijerph22020301 (PMC11855579; doi:10.3390/ijerph22020301)
Supplement: Supplementary file 1 [file ijerph-22-00301-s001.zip › ijerph-3304244-supplementary.pdf]

Supplementary Material 1

Section (1) Additional health economic questions

1. On a scale of 0 to 10 (where 0 is no change and 10 is maximum change) how much do you feel that any improvement in your health and wellbeing:

(a) is due to using the Citizens Advice CAP service?

(b) would have happened anyway even if you hadn't used the Citizens Advice CAP service?

The answers to (a) and (b) should add up to a total of 10

2. Did attending the Citizens Advice CAP service mean you had to cut back on other activities that benefited your health and wellbeing?

Prompt: If participant answers "yes" please ask "How much did this effect these activities:

a small amount a moderate amount a large amount a very large amount

3. How long do you feel that any changes due to attending the Citizens advice service will last:

less than a month 3 months 6 months a year longer than a year

Supplementary Material 3 **Responses to additional health economic questions**

Service User Code	Collected From	% Change in Wellbeing Due to CAP (Attribution)	% Change in Wellbeing Due to Other Factors	Impact on Other Factors That Impact Wellbeing (Displacement)	Expected Length of Service Impact (Drop-off)
1	interview	n/a	n/a	a small amount	n/a
2	interview	90%	10%	no impact	over 12 months
3	interview	50%	50%	no impact	6–12 months
4	interview	50%	50%	no impact	1 to 3 months
5	interview	70%	30%	no impact	over 12 months
6	interview	70%	30%	no impact	over 12 months
7	interview	80%	20%	no impact	over 12 months
8	interview	80%	20%	no impact	6–12 months
9	interview	90%	10%	no impact	over 12 months
**Average % of interviews**		**72.5%**	**27.5%**		
10	PN focus group	50%	50%	no impact	over 12 months
11	PN focus group	70%	30%	no impact	over 12 months
12	PN focus group	n/a	n/a	no impact	n/a
13	PN focus group	30%	70%	no impact	3 to 6 months
14	PN focus group	60%	40%	no impact	over 12 months
15	PN focus group	70%	30%	no impact	over 12 months
16	PN focus group	80%	20%	no impact	6 to 12 months
**Average % PN focus group**		**60.0%**	**40.0%**		

PN = perinatal; n/a = In two of the sixteen qualitative interviews, the interviewing team did not ask questions about attribution, displacement, and drop-off
